# A bleeding score identifies who bleeds, not who benefits: anticoagulation decisions in post-operative atrial fibrillation after coronary surgery

**DOI:** 10.1093/ehjcvp/pvag049

**Published:** 2026-07-02

**Authors:** Xiang Zhou, Hongyan Liu, Zhengdong Hua

**Affiliations:** Department of Cardiac Surgery, Wuhan Asia Heart Hospital, Wuhan University of Science and Technology, No. 753 Jinghan Avenue, Jianghan District, Wuhan 430022, China; Wuhan Clinical Center for Cardiomyopathy, No. 753 Jinghan Avenue, Jianghan District, Wuhan 430022, China; Department of Cardiac Surgery, Wuhan Asia Heart Hospital, Wuhan University of Science and Technology, No. 753 Jinghan Avenue, Jianghan District, Wuhan 430022, China; Wuhan Clinical Center for Cardiomyopathy, No. 753 Jinghan Avenue, Jianghan District, Wuhan 430022, China; Department of Cardiac Surgery, Wuhan Asia Heart Hospital, Wuhan University of Science and Technology, No. 753 Jinghan Avenue, Jianghan District, Wuhan 430022, China; Wuhan Clinical Center for Cardiomyopathy, No. 753 Jinghan Avenue, Jianghan District, Wuhan 430022, China

Rezk and colleagues^1^ show that a four-item PRECISE-DAPT score stratifies 1-year post-discharge major bleeding (0.8%, 1.9%, and 3.6% across low-, moderate-, and high-risk groups) in 4436 patients with new-onset atrial fibrillation (AF) after coronary artery bypass grafting who received no early oral anticoagulation (OAC). They suggest that, in high-risk patients ‘with sinus rhythm at discharge and a normal stroke risk (i.e. CHA_2_DS_2_-VASc score of 3–4), it appears reasonable to shorten the period of OAC or withhold it unless AF recurs.’ Our concern is one of principle. A bleeding score identifies who is likely to bleed, not who benefits from anticoagulation. Whether to give or withhold OAC turns on net clinical benefit—strokes prevented set against bleeds caused—and a bleeding score measures only one side of that balance. Three features of the authors’ own data show why this single-sided tool cannot carry the two-sided decision they propose.

First, the recommendation's own stroke condition is at odds with the authors’ data. It rests on labelling a CHA_2_DS_2_-VASc of 3–4 as a ‘normal’ stroke risk, yet 3–4 is itself a guideline indication for OAC, so withholding anticoagulation from this subgroup runs against the very threshold that defines it. The high-bleeding-risk group as a whole averaged a CHA_2_DS_2_-VASc of 4.7 ± 1.5 (median 5), so the ‘normal-risk’ descriptor sits well below the stroke risk of the population the recommendation addresses. Consistent with this, post-discharge ischaemic stroke was not lower—if anything numerically higher—in the high-bleeding-risk group (1.1%, 1.2%, and 1.4% across low, moderate, and high risk): bleeding and stroke risk were not inversely related, and the patients flagged to forgo anticoagulation were not those at lower stroke risk. The magnitude of anticoagulation's benefit in this low-burden, post-discharge setting is itself debated—the authors cite a meta-analysis of more bleeding without a thromboembolic reduction—but that uncertainty underscores rather than resolves the problem: when the stroke side of the balance is unsettled, a tool that measures only bleeding cannot drive the decision in either direction.

Second, the bleeding side of the balance is overestimated exactly where the recommendation acts. The score ‘overestimated bleeding risk in patients with a predicted risk >7%,’ and the authors themselves note that this ‘may lead to underuse of OAC in patients who might still benefit.’ That is precisely the harm our concern identifies: the high-risk label used to justify withholding OAC is inflated in the very stratum where the decision is taken—by the authors’ own admission—yet the recommendation follows the inflated estimate rather than their caveat.

Third, even on the authors’ own utility analysis, the score's benefit does not extend to the decision it is invoked for. Their decision-curve analysis shows net benefit only for bleeding-probability thresholds of roughly 1% to 4.5%, beyond which it ‘approaches that of the ‘treat none’.’ Withholding stroke-preventing OAC, however, is a decision a clinician would reach only at a substantially higher bleeding-risk threshold—precisely the range in which the score, by this analysis, adds nothing. The tool is also moved beyond its basis: PRECISE-DAPT predicts bleeding on antiplatelet therapy,^2^ and is applied here to a different exposure (OAC) and a different decision (whether to anticoagulate, rather than how long to continue dual antiplatelet therapy).

We share the authors’ caveat that the study ‘does not prove that using this score to guide anticoagulation improves clinical outcomes.’ Our point is narrower and follows from their own figures: shortening or withholding OAC in the high-bleeding-risk group would act on patients with a clear guideline anticoagulation indication and a stroke risk no lower than their peers’, at bleeding thresholds where the score adds no net benefit and overestimates risk. A bleeding score is a necessary input to the anticoagulation decision; it cannot, by itself, identify who should not be anticoagulated.

## Data Availability

No new datasets were generated or analysed in this correspondence. All information discussed is available in the published source article cited in the reference list.
